# Dissociating Profiles of Social Cognitive Disturbances Between Mixed Personality and Anxiety Disorder

**DOI:** 10.3389/fpsyg.2020.00563

**Published:** 2020-03-26

**Authors:** Kristína Czekóová, Daniel Joel Shaw, Zuzana Pokorná, Milan Brázdil

**Affiliations:** ^1^Behavioral and Social Neuroscience Research Group, CEITEC, Masaryk University, Brno, Czechia; ^2^Institute of Psychology, Czech Academy of Sciences, Brno, Czechia; ^3^Department of Psychology, School of Life and Health Sciences, Aston University, Birmingham, United Kingdom; ^4^Department of Psychiatry, Faculty of Medicine, Masaryk University Brno and University Hospital, Brno, Czechia

**Keywords:** Social cognition, personality disorder, anxiety, emotion recognition, imitative control, visual perspective taking, empathy, emotion regulation

## Abstract

**Background:**

An emerging body of research has begun to elucidate disturbances to social cognition in specific personality disorders (PDs). No research has been conducted on patients with Mixed Personality Disorder (MPD), however, who meet multiple diagnostic criteria. Further, very few studies have compared social cognition between patients with PD and those presenting with symptomatic diagnoses that co-occur with personality pathologies, such as anxiety disorder (AD). The aim of this study was to provide a detailed characterization of deficits to various aspects of social cognition in MPD and dissociate impairments specific to MPD from those exhibited by patients with AD who differ in the severity of personality pathology.

**Method:**

Building on our previous research, we administered a large battery of self-report and performance-based measures of social cognition to age-, sex- and education-matched groups of patients with MPD or AD, and healthy control participants (HCs; *n* = 29, 23, and 54, respectively). This permitted a detailed profiling of these clinical groups according to impairments in emotion recognition and regulation, imitative control, low-level visual perspective taking, and empathic awareness and expression.

**Results:**

The MPD group demonstrated poorer emotion recognition for negative facial expressions relative to both HCs and AD. Compared with HCs, both clinical groups also performed significantly worse in visual perspective taking and interference resolution, and reported higher personal distress when empathizing and more state-oriented emotion regulation.

**Conclusion:**

We interpret our results to reflect dysfunctional cognitive control that is common to patients with both MPD and AD. Given the patterns of affective dispositions that characterize these two diagnostic groups, we suggest that prolonged negative affectivity is associated with inflexible styles of emotion regulation and attribution. This might potentiate the interpersonal dysfunction exhibited in MPD, particularly in negatively valenced and challenging social situations.

## Introduction

Disturbances to interpersonal functioning are recognized increasingly as a characteristic symptom of many psychiatric conditions, particularly personality disorders (PD; Schilbach, 2016; Cotter et al., 2018). This is reflected in the redefinition of personality pathology in the most recent version of the Diagnostic and Statistical Manual (DSM-5); in addition to impairments in cognition, affectivity and impulse control, interpersonal dysfunction is considered a primary manifestation of personality pathologies (Hopwood et al., 2013). Such interpersonal dysfunction will result in altered interaction styles that impact negatively on the quality of social exchanges, thereby reinforcing patients’ maladaptive patterns of thought and behavior in a self-perpetuating manner (Schilbach, 2016). This can be observed in the clinical setting; the interpersonal difficulties characterizing PD have negative effects on patient-clinician interactions, thereby compromising the efficacy of therapy and increasing the risk of chronicity (Tyrer et al., 2015). In this light it is essential to develop effective interventions for the interpersonal dysfunction shown in PD, but this demands a precise characterization of the impairments shown by these patients.

The broad repertoire of cognitive and affective abilities that support efficient social interaction and enable adaptive behavior within social environments are referred to collectively as *social cognition*. Despite a lack of agreement concerning the structure of cognitive skills subsumed within this psychological construct, and considerable variability among existing taxonomies (Happé et al., 2017), several facets have been investigated in both healthy and clinical populations: (1) Emotion recognition – our ability to recognize emotional expressions in others; (2) imitative control – the capacity to inhibit our automatic tendency to imitate the actions of others; (3) visual perspective taking – the process through which we infer what others can see; (4) empathy – our ability to understand and share in the emotions of others (cognitive and affective empathy, respectively), whilst recognizing that they are the source of one’s own emotional state (Cuff et al., 2014); and (5) emotion regulation – the capacity to self-regulate our emotional responses in order to behave appropriately in the face of changing environmental demands (Aldao, 2013). These distinct facets of social cognition are assumed to be organized in a hierarchical manner, whereby complex abilities build on more elementary mechanisms – efficient emotion recognition and imitative control are suggested to be necessary for affective empathy, for example, and low-level perspective taking is considered a prerequisite for cognitive empathy (Decety, 2011; Happé et al., 2017; Shaw et al., 2018). Another fundamental cognitive mechanism believed to serve a crucial role in all of the aforementioned processes is one that allows us to distinguish and switch flexibly between self- and other-representations. Such self-other distinction is necessary for self-regulation during social interactions; it will allow us to control imitative tendencies, to avoid personal distress when empathizing by attributing the cause of our own emotional responses to the target of our empathic expression, and to prevent the misattribution of our own egocentric viewpoint onto others when inferring their perspective (Decety, 2011; Lamm et al., 2016; Steinbeis, 2016).

Research into these socio-cognitive abilities in PD have begun to reveal impairments in many diagnostic groups. This includes disturbances in the discrimination of emotional facial expressions in Narcissistic (Marissen et al., 2012), Antisocial (Zhang et al., 2016) and Borderline PD (Berenson et al., 2018); reduced imitative control in Borderline PD (Hauschild et al., 2018); an inability to accurately infer the mental states of others in Narcissistic (Bilotta et al., 2018) and Avoidant PD (Moroni et al., 2016); subtle alterations in empathic awareness and expression in Obsessive–Compulsive (Cain et al., 2015) and Narcissistic PD (Baskin-Sommers et al., 2014); and differential patterns of dysfunctional emotion regulation among all Cluster A, B and C diagnoses (Borges and Naugle, 2017; for reviews on social cognition, see Herpertz and Bertsch, 2014; Cotter et al., 2018). Interestingly, in Borderline PD these impairments have been attributed to dysfunctional self-other distinction (Beeney et al., 2016). While this research has begun to provide valuable insights into the nature of socio-cognitive disturbances emerging in specific forms of personality pathology, the focus on discrete diagnostic categories has meant that patients presenting with symptoms that span multiple diagnostic criteria have received very little attention. This is concerning given the high prevalence of the Mixed PD category (MPD) in clinical practice (World Health Organization, 2004; Zimmerman et al., 2005). Moreover, it is suggested that a categorical diagnostic system is inappropriate for PD given the vast heterogeneity exhibited by patients; artificially discrete diagnoses ignore the comorbidities shared among different diagnostic groups (Zimmerman et al., 2005; Clark et al., 2015; Tyrer et al., 2015).

Given the issues associated with the categorical model of PD, a considerable number of clinicians favor alternative hybrid or dimensional models (Morey and Hopwood, 2019). The dimensional approach places personality on a continuum ranging from personality style to disorder; PD is considered to sit on the latter pathological end of this continuum, characterized by a limited repertoire of personality styles that are used inflexibly in response to changing environmental demands. From this dimensional perspective, similar but less severe profiles of socio-cognitive disturbances should be observed in healthy individuals who exhibit the same pattern of inflexibility among certain personality styles as PD patients (e.g., emotional instability). Indeed, in a previous investigation conducted on a large healthy sample we revealed associations between individual differences in the flexible deployment of personality styles and various components of social cognition: Relative to individuals reporting a broader repertoire of personality styles, those showing a strong preference for avoidant, borderline, depressive, and dependent styles exhibited less control over imitative tendencies, maladaptive styles of emotion regulation, and greater distress when empathizing with others (Shaw et al., 2018). This suggests that personality mechanisms giving rise to maladaptive tendencies toward specific personality traits might drive the patterns of social cognitive dysfunction observed in PD.

The dimensional approach to PD could also elucidate other important mechanisms behind social cognitive dysfunction; for example, difficulties in specific socio-cognitive abilities might be driven by symptoms such as social withdrawal and emotional instability that result from the altered personality emerging across multiple PDs (Hopwood et al., 2011; Semerari et al., 2014, 2015; Moroni et al., 2016). Importantly, many discrete PD diagnostic groups display high comorbidity with anxiety and depression (Friborg et al., 2013; Kret and Ploeger, 2015), particularly Cluster C pathologies such as Avoidant, Dependent and Obsessive-Compulsive PD (Friborg et al., 2013). Although previous research indicates that disturbances in social cognition might differentiate between PD and emotional (e.g., anxiety) disorders (Semerari et al., 2014), very few studies have contrasted social cognition among these patient groups (Pellecchia et al., 2018).

The aim of this study was to provide a detailed characterization of disturbances to social cognition in MPD, and to dissociate these impairments according to severity of personality pathology. To do so, we compared performance on a large test battery comprising various measures of social cognition among groups of age- and sex-matched, patients with MPD, patients with anxiety disorder (AD), and healthy control participants (HCs). Both patient groups were characterized by anxiety and depressive symptoms, but differed in the severity of personality pathology; specifically, the degree of disturbance to interpersonal functioning. This allowed us to identify impairments to social cognition specific to MPD or common to both psychiatric groups. Given our previous finding that personality-related individual differences in social cognition span multiple levels (Shaw et al., 2018), we measured socio-cognitive abilities believed to reflect both fundamental mechanisms as well as higher-level processes; specifically, the test battery included measures of emotion recognition, imitative control and visual perspective taking, with tests of higher-level cognitive and affective empathy, and emotion regulation. Our choice of specific measures was driven by research in both healthy and clinical samples, such as patients with autism spectrum disorder (Sowden et al., 2016; Deschrijver et al., 2017), schizophrenia (Langdon et al., 2001), depression (Grunewald et al., 2015) and multiple sclerosis (Czekóová et al., 2019).

On the basis of the previous research summarized above, we predicted that the greater severity of personality disturbances presented by MPD patients would manifest as poorer discrimination among emotional facial expressions, less control over imitative tendencies, worse perspective taking, reduced empathic expression, and altered emotion regulation relative to those with AD and HCs.

## Materials and Methods

### Sample

The sample comprised 29 patients diagnosed with MPD and 23 with anxiety disorder (AD) recruited from University Hospital, Brno, Czechia from 2016 to 2018, and 54 healthy control (HC) participants matched on age, sex, and education (highest attainment). The HC sample was selected among staff and associates of Masaryk University who reported no history (or presence) of psychiatric or neurological disorders, periods of emotional dysfunction, participation in psychotherapy, or psychiatric medication. All patients were admitted at their own request, and diagnosed independently by a psychiatrist and a psychologist at the beginning of their treatment according to the International Personality Disorders Examination (Loranger et al., 1997). The MPD group comprised patients meeting diagnostic criteria of multiple PDs, the most frequent of which were histrionic, borderline, avoidant, and narcissistic PDs. The second group comprised patients who met criteria for anxiety disorder (AD), including social and generalized anxiety, somatization, mixed anxiety-depressive, and panic disorder. Importantly, patients with Posttraumatic Stress Disorder were not included in this sample due to known differences in their socio-cognitive disturbances compared with other anxiety diagnoses (Plana et al., 2014). The two clinical groups did not differ with respect to anxiety or depressive symptoms. Patients and HCs were included only if they reported no past or present neurological disorders (e.g., epilepsy), substance dependence, or psychotic disorders. None of the patients were confirmed to suffer from migraine. For details on demographic and clinical characteristics of the sample, see Table 1.

**TABLE 1:** Sample demographics and clinical characteristics.

			Groups					
			MPD		AD	HCs		*p*
Age (mean [SE])			34.6 (2.3)		38.0 (2.3)	34.6 (1.7)		0.497
Sex (male/female frequency)			5/24		7/16	14/40		0.505
Education (median years [IQR])			13 (11–13)		13 (11–18)	13 (13–18)		0.091
Anxiety (%)			93.1		100	–		0.497
Depressive symptoms (%)			89.7		82.6	–		0.686

**Percentage of diagnostic comorbidity MPD**								

**HIS**	**EMU**	**AVO**	**NAR**	**DEP**	**ANA**	**SCH**	**PAR**	**DIS**

48.3	41.4	37.9	31.0	27.6	20.7	13.8	10.3	0.0

**Percentage of diagnostic comorbidity AD**								

**MAD**		**GAD**		**SOM**		**PAD**		**SOC**

47.8		30.4		26.1		17.4		13.1

*MPD, Mixed Personality Disorder; AD, Anxiety Disorder; HCs, Healthy Controls; IQR, Inter-quartile range; HIS, Histrionic; EMU, Emotionally Unstable; AVO, Avoidant; NAR, Narcissistic; DEP, Dependent; ANA, Anankastic; SCH, Schizoid; PAR, Paranoid; DIS, Dissocial; MAD, Mixed Anxiety and Depressive Disorder; GAD, Generalized Anxiety Disorder; SOM, Somatoform Disorder; PAD, Panic Disorder; SOC, Social Phobia.*

The study was approved by the Ethics Board of the Institute of Psychology, Academy of Sciences in the Czechia, and all participants provided written informed consent prior taking part.

### Procedure

A large battery of performance-based (implicit) and self-report (explicit) measures of social cognition was administered to all participants. This battery was developed in a previous study (Shaw et al., 2018) to assess state affectivity at the time of testing, imitative control, emotion recognition, empathic awareness and expression, low-level visual perspective taking, and emotion regulation. In the section below, each test comprising this battery is described briefly in the order they were administered. The reader is referred to our original paper for more details.

All measures comprising the test battery were programed and presented in Cogent (v1.31)^1^, a toolbox for MATLAB (vR2015b; The MathWorks Inc., Natick, MA, United States). Completion of the test battery took approximately 1.5 h.

#### Affective State

To examine whether participants’ positive and negative affectivity at the time of testing differed between the study groups, which might confound other measures of social cognition, we administered the Implicit Positive and Negative Affect Test (Quirin et al., 2009). This measure achieves good test-retest reliability and construct validity, and has been found to predict physiological indices of affectivity more accurately than explicit self-report instruments (Quirin et al., 2009; Quirin and Bode, 2014).

This task involved six artificial stimulus words (e.g., “SAFME,” “TUNBA”). Using a 4-point Likert scale (1 = *“Does not fit at all”*, 4 = *“Fits very well”*), participants rated the extent to which these words convey six different mood states (happy, cheerful, energetic, helpless, tense or inhibited). Each artificial word was presented with one of the six mood-state adjectives in a pseudo-random order, resulting in 36 trials. Ratings for positive and negative mood states were averaged and used as the dependent measures.

#### Emotion Recognition

To assess participants’ ability to discriminate among neutral and emotionally evocative stimuli, the battery included an emotional Go/No-Go task (e.g., Tottenham et al., 2011). This task comprised 6 blocks of 40 trials, each block presenting a pseudo-randomized sequence of face stimuli with either emotional or neutral expression. Each trial started with a fixation cross that was followed immediately by 500 ms presentation of a face. Participants were instructed to press the spacebar as quickly as possible whenever the face portrayed a particular expression (“Go” trials), and to withhold that action when a different expression was shown (“No-go” trials). A pre-potent tendency to respond was induced by the ratio of the two trial types (Go = 70%, No-go = 30%). No more than two No-go trials were presented in succession within a block. In any given block, an emotional expression was always paired with a neutral expression; the emotional expression was either the “Go” stimulus (with the neutral expression as the “No-Go” stimulus) or the “No-Go” stimulus (with the neutral expression serving as the “Go” stimulus). The task comprised three emotional (angry, fearful, and happy expressions) and three non-emotional blocks (neutral expressions). A short practice block was administered for participants to familiarize themselves with the task. The stimuli were selected from the Radboud Faces Database (14 males; Langner et al., 2010), gray scaled and cropped to remove any hair.

Performance was indexed by D-prime scores, calculated separately for each individual block by subtracting the z-transformed false alarm rate from the z-transformed hit rate for both stimulus types. As such, higher scores on each block indicate better discrimination between the Go and No-go emotions.

#### Imitative Control

Imitation was examined implicitly with a Stimulus-Response Compatibility task (Brass et al., 2001; Cracco et al., 2018), whereby participants execute finger-lifting actions in response to a colored dot (imperative stimulus) while observing task-irrelevant finger actions performed by a model’s hand (stimulus hand). On this task, executed finger movements are found consistently to be faster and more accurate in response to matching (compatible) compared with opposing (incompatible) observed movements. This is referred to as automatic imitation, and is used as an experimental measure of imitative control.

Each trial began with the stimulus hand resting on a flat surface, whereby participants depressed both the left and right directional arrows on a standard keyboard with the index and middle finger of their right hand, respectively. After a random interval of 800–2400 ms, this warning stimulus was replaced with an end-point image of the same stimulus hand performing either an index or middle finger extension. This end-point image contained a dot located between the index and middle finger, the color of which indicated whether the participant should extend their own index or middle finger as quickly as possible. The inter-trial interval was 1000 ms. Two blocks of 90 trials were administered: 30 Compatible (the same action was both signaled and observed), 30 Incompatible (different actions were signaled and observed), 20 Baseline (a movement was signaled but not observed), and 10 Catch trials (no action was signaled, but a movement was observed). Five practice trials were completed before the task began.

Employing the same approach taken in other studies (for reviews see Heyes, 2011; Cracco et al., 2018), the difference in response time (RT) on Incompatible relative to Compatible trials was used as our measure of interest – a greater compatibility effect was taken to index less control over imitative tendencies. Genschow et al. (2017) reported a high split-half reliability of 0.86 for this compatibility effect. Importantly, each block presented the model’s hand in either a clockwise (+90°), or counter-clockwise rotation (−90°) from the participants’ perspective. This allowed us to isolate imitative compatibility from the potentially confounding influences of spatial-compatibility effects (for illustrations see Shaw et al., 2017). To separate the sources of automatic imitation, we calculated RTs in each block separately.

#### Visual Perspective Taking

Level-1 visual perspective taking was investigated with The Director Task (e.g., Keysar et al., 2000), whereby participants move objects around a grid of shelves according to verbal instructions given by a “Director.” This task has been used elsewhere to investigate both the developmental trajectory (Dumontheil et al., 2010) and neurophysiological underpinnings of visual perspective taking (for a review see Bukowski, 2018). Further, this task achieved excellent split-half reliability in our previous study (Shaw et al., 2018). Some of the boxes have opaque backs, which means that the Director cannot see their contents from her position behind the shelf display – these are visible only from participant’s vantage point. The Director’s instruction on experimental trials refers to an object placed in one of the opaque boxes (e.g., “*Move the smallest apple*”), creating a discrepancy between the Director and participants’ perspectives. To follow the instructions correctly, the participant must ignore their own perspective and discount any “distractor” objects not visible to the Director (e.g., they were to move the medium-sized apple rather than the smallest). In three different control conditions, no distractor object is present to create a discrepancy: In the first it is replaced with a different object, in the second the Director’s instructions refer to objects within boxes without opaque backs, and in the third the Director is absent and participants must carry out instructions according to their own perspective.

Participants responded by indicating with the mouse into which box the object should be moved, and RT and accuracy was measured on each trial. To allow for comparisons with our previous findings, as an index visual perspective-taking ability we calculated relative measures of both RT and accuracy by regressing averaged values across all control conditions from those in the experimental condition (see Shaw et al., 2017; 2018). This produced standardized residuals for each participant, with higher values representing longer RTs and higher accuracy on experimental relative to control trials. Since correlational analyses between RT and accuracy showed no sign of a speed-accuracy trade-off (*p* ≥ 0.126), inverted efficiency scores were not calculated.

#### Empathy

##### Implicit measure

To measure empathy implicitly, we used a task developed previously (Shaw et al., 2018) that follows the same principles as the Multifaceted Empathy Test to delineate between cognitive and affective components (Dziobek et al., 2008). The task involved two blocks of 30 color photographs depicting individuals in different emotional contexts. Each photograph was presented randomly in each block for a maximum of 10 s. Participants were required to (1) select one of four emotion labels that best describes how the person in the image is feeling (cognitive empathy), and to (2) rate their own arousal while observing the image on a 7-point Likert scale (1 = *“None,”* 7 = *“Very strong”*; affective empathy).

Importantly, since cognitive empathy did not achieve acceptable reliability in our previous large-scale sample (Shaw et al., 2018), we decided to focus only on arousal ratings as a measure of affective empathy herein.

##### Explicit measure

The Interpersonal Reactivity Index (Davis, 1983) was employed as a multidimensional self-report measure of cognitive and affective aspects of trait empathy. This instrument has been shown to be both valid and highly reliable across European samples (e.g., De Corte et al., 2007; Gilet et al., 2013). Cognitive facets are represented by the subscales Perspective Taking (the motivation/tendency to adopt the psychological perspective of others spontaneously) and Fantasy (the tendency to transpose oneself imaginatively into the feelings and actions of fictional characters). Affective empathy is indexed by Empathic Concern (the tendency to experience feelings of sympathy and concern toward others) and Personal Distress (the tendency to experience personal anxiety and unease in tense interpersonal settings). This instrument comprises 28-items, each rated on a five-point Likert scale (*1* = *“Does not describe me well,” 5* = *“Describes me very well”*).

#### Emotion Regulation

The Action Control Scale (Kuhl and Beckmann, 1994) was employed to assess participants’ self-reported ability to regulate their negative and positive affective states in a flexible and adaptive manner under different conditions. Scores indicate the degree to which an individual has this capacity for action-oriented emotion regulation in two domains: Failure vs. preoccupation (AOF) and Demand vs. hesitation (AOD). Importantly, both of these sub-scales achieved acceptable reliability in our previous large-scale study (Shaw et al., 2018). Each subscale provides two alternatives in response to 12 everyday situations; for instance, in response to the statement “*When several things go wrong on the same day*,” participants can choose a state-oriented (“*I don’t know how to deal with it”*) or action-orientation response (“*I just keep on going as though nothing had happened”*). Low scores indicate state-oriented tendencies toward negative affect – that is, the tendency to ruminate over past failures and limited self-access (AOF), and a limited ability to preserve positive affect in complex and difficult situations (AOD).

## Results

**FIGURE 1:**
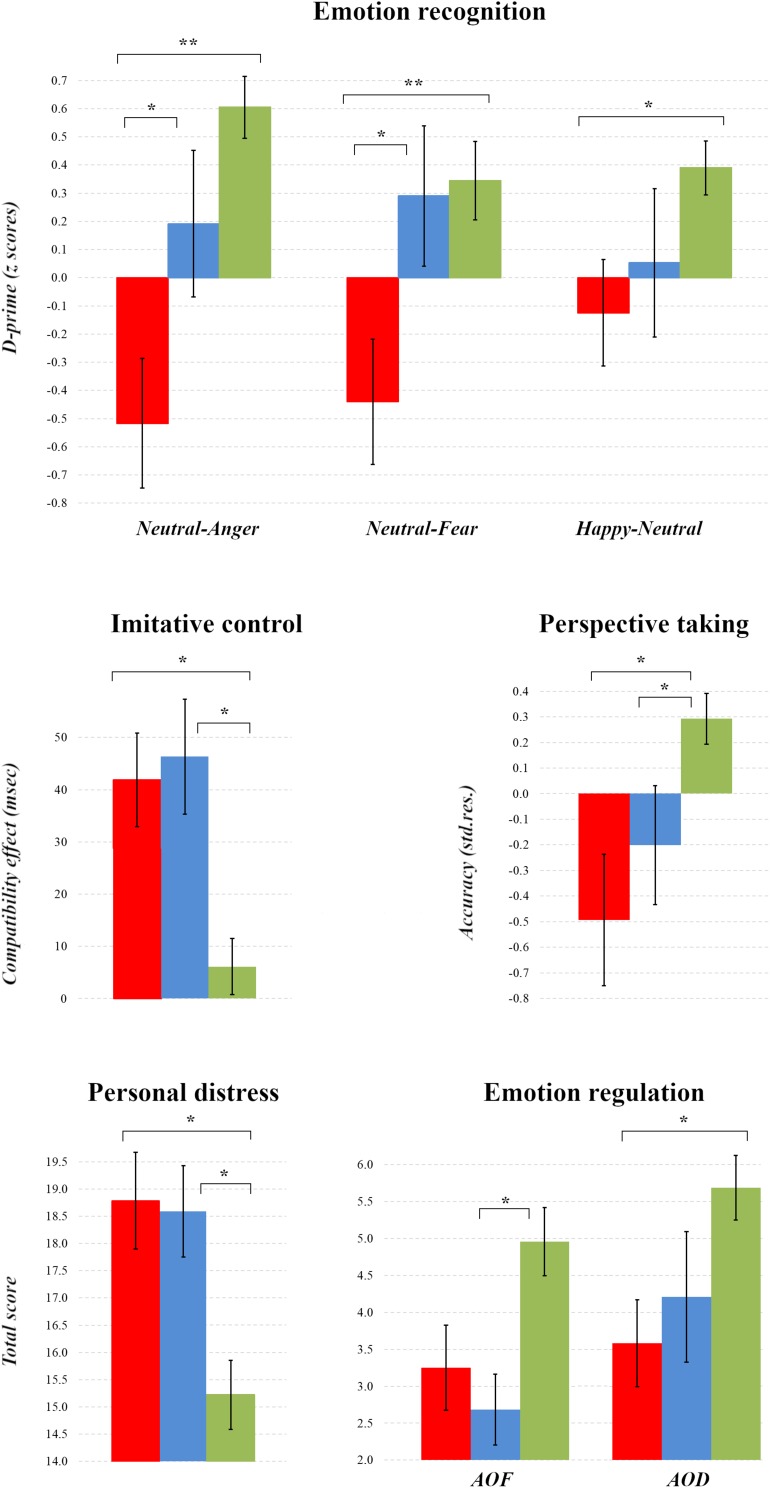
Comparisons of Socio-cognitive Aspects among Patient Groups and Healthy Controls. For comparisons with other studies, plots present means and standard errors for the MPD (*red*), AD (*blue*) and HC groups (*green*). ^∗∗^*p* < 0.01, ^∗^*p* < 0.05.

Since the majority of variables did not meet the assumptions of parametric testing, performance among the groups was compared using non-parametric Kruskal-Wallis and follow-up Mann-Whitney tests corrected for multiple comparisons with the false discovery rate (Benjamini and Hochberg, 1995). Table 2 presents the group medians and interquartile ranges for measures with comparable performance, and Figure 1 illustrates all significant differences among the groups.

**TABLE 2:** Descriptives of non-differentiating variables.

Variable		MPD	AD	HCs
Affect	Positive	2.06(1.78−2.50)	2.17(1.83−2.53)	2.19(1.72−2.44)
	Negative	2.28(1.81−2.39)	2.22(1.90−2.60)	2.06(1.88−2.44)
Imitation		−2.44(−32.48−23.28)	−9.82(−23.07−12.26)	3.23(−15.94−21.49)
Emotion recognition	A-N	0.06(−1.16−0.95)	0.57(−0.64−1.23)	0.51(−0.40−0.96)
	F-N	−0.20(−1.25−0.71)	0.32(−0.73−1.01)	0.62(−0.22−1.31)
	N-H	0.29(−0.43−0.82)	0.65(−0.45−1.00)	0.47(−0.39−1.18)

*Values present medians (and interquartile ranges). MPD, Mixed Personality Disorder; AD, Anxiety Disorder; HCs, Healthy Controls; Imitation, imitative control in response to the stimulus isolating imitative from spatial compatibility (see text); Emotion recognition, D-prime scores (z-transformed) on angry Go/neutral No-go (A-N); fearful Go/neutral No-go (F-N); and neutral Go/happy No-go blocks.*

The MPD, AD, and HC groups did not differ on the implicit measure of positive (X[2] = 0.382, *p* = 0.826) or negative affective state at the time of testing (X[2] = 1.503, *p* = 0.472).

On the Stimulus-Response Compatibility procedure, the three groups exhibited statistically equivalent imitative control in response to the stimuli that isolated imitative compatibility from confounding spatial influences (X[2] = 1.391, *p* = 0.499). In response to the stimulus affording confounding spatial-compatibility effects, however, there were significant differences among the groups (X[2] = 13.302, *p* = 0.001), whereby both the MPD and AD groups expressed significantly greater compatibility effects relative to the HC group (*Z* = −2.999, *p*_*corr.*_ = 0.005, *r* = 0.33, 95% CI [−0.11, −0.55]; *Z* = −2.923, *p*_*corr.*_ = 0.005, *r* = 0.34 [−0.11, −0.56]), but did not differ from one another (*p*_*corr.*_ > 0.05).

On the Go/No-Go task, a comparison of D-prime scores in each block revealed that the three groups differed only in their ability to discriminate neutral from angry (X[2] = 15.905, *p* < 0.001) and fearful (X[2] = 10.004, *p* = 0.007) No-go facial expressions, and happy from neutral No-go expressions (X[2] = 7.101, *p* = 0.029). Follow-up tests revealed that all these differences reflected poorer discriminatory ability in the MPD group relative to HCs for the angry (*Z* = −4.01822, *p*_*corr.*_ < 0.001, *r* = 0.45 [−0.23, −0.68]), fearful (*Z* = −3.090, *p*_*corr.*_ = 0.006, *r* = 0.35 [−0.12, −0.57]) and happy blocks (*Z* = −2.719, *p*_*corr.*_ = 0.018, *r* = 0.30 [−0.08, −0.52]), and in the MPD compared with the AD group on blocks containing angry (*Z* = −2.206, *p*_*corr.*_ = 0.041, *r* = 0.31 [−0.03, −0.59]) and fearful expressions (*Z* = −2.273, *p*_*corr.*_ = 0.033, *r* = 0.32 [−0.04, −0.60]).

In terms of empathy, the three groups did not differ significantly from one another on the implicit measure of arousal (X[2] = 4.265, *p* = 0.119), nor in their self-reported Perspective Taking (X[2] = 1.926 *p* = 0.382), Empathic Concern (X[2] = 4.842, *p* = 0.089), or Fantasy (X[2] = 3.060, *p* = 0.217). The groups did differ, however, in their self-reported levels of Personal Distress when empathizing (X[2] = 12.776, *p* = 0.002), with both clinical groups scoring equivalently higher than the HCs (MPD: *Z* = −3.030, *p*_*corr.*_ = 0.006, *r* = 0.33 [−0.12, −0.55]; AD: *Z* = −2.735, *p*_*corr.*_ = 0.009, *r* = 0.31 [−0.09, −0.54]).

Group comparisons of perspective-taking performance on experimental relative to control trials in the Director Task revealed that all three groups responded correctly with equivalent RTs (X[2] = 4.391, *p* = 0.111), but differed in their accuracy (X[2] = 7.842, *p* = 0.020); specifically, both clinical groups achieved poorer accuracy relative to the HCs (MPD: *Z* = −2.323, *p*_*corr.*_ = 0.045, *r* = 0.26 [−0.04, −0.49]; AD: *Z* = −2.164, *p*_*corr.*_ = 0.045, *r* = 0.26 [−0.02, −0.49]), but did not differ significantly from each other (*p*_*corr.*_ = 0.947).

Finally, the groups differed in failure- (AOF: X[2]) = 9.629, *p* = 0.008) and demand-related emotion regulation (AOD: X[2] = 8.955, *p* = 0.011); while the AD group reported more state-oriented emotion regulation following failure relative to the HCs (*Z* = −2.892, *p*_*corr.*_ = 0.009, *r* = 0.35 [−0.11, −0.59]), the MPD participants reported more demand-related state orientation compared to HCs (Z = −2.816, *p*_*corr.*_ = 0.012, *r* = 0.33 [−0.10, −0.56]).

## Discussion

The aim of this study was to provide a detailed, multi-level characterization of disturbances to social cognition in patients with Mixed Personality Disorder (MPD) relative to patients with anxiety disorder (AD), and healthy controls (HCs). To achieve this, we administered a battery of tests measuring both high- and low-level socio-cognitive abilities to all three groups, which has been shown to dissociate among personality styles differing in emotional dispositions (Shaw et al., 2018). Our results reveal discrete patterns of dysfunctional social information processing in both clinical groups compared with HCs, but more severe socio-cognitive impairments in patients with MPD. In the discussion below, we interpret each of the principle findings.

Firstly, patients with MPD demonstrated poorer discrimination of certain emotional facial expressions relative to the HC; this was true for both angry and fearful faces presented within a neutral context, and neutral faces within a prevailing context of happy expressions. This finding both supports and extends previous research into PD: Poorer accuracy in the classification and identification of fearful faces has been reported in patients with Avoidant, Narcissistic, and Borderline PD (Rosenthal et al., 2011; Marissen et al., 2012; Semerari et al., 2014), and a lower sensitivity to anger and happiness has been observed in non-clinical individuals with avoidance problems (Vrijen et al., 2016). In MPD, this impairment in emotion processing appears to go beyond the general response bias toward negatively valenced emotions reported elsewhere (Mitchell et al., 2014; Semerari et al., 2014). More importantly, the poorer performance of these patients on blocks in which pre-potent responses were to be withheld for negative facial expressions appears to be related to the severity of personality pathology; the MPD group performed worse on these blocks than patients with AD.

Given the other results of this study discussed below, this seemingly specific impairment reveals important differences between the MPD and AD groups. The GNG task requires participants to perform a number of cognitive operations at speed – they must identify the emotion expressed by a given face, recall whether or not a response is required, and, on No-go trials, inhibit an inappropriate action. This places huge demands upon cognitive control processes – namely, the monitoring and updating of working memory representations, switching flexibly between multiple tasks, and the intentional overriding of a dominant or pre-potent response (see Miyake et al., 2000; Diamond, 2013). Poorer performance on this measure of social cognition in the MPD relative to AD group may therefore indicate that the former patients experience greater dysfunction in cognitive control under speeded discrimination among negative and neutral facial expressions. In the same way that this impedes performance on a socio-cognitive task, such dysfunction would hamper social exchanges and serve to exacerbate symptoms of dysfunctional social interaction, especially when it involves negative emotions.

At first glance, both the MPD and AD patient groups appear to exhibit significantly poorer control over imitative tendencies compared with HCs – they were slower at executing actions while they observed incompatible compared with compatible actions performed by another individual, which is taken as an experimental index of mimicry (Heyes, 2011). Importantly, however, differences between the clinical and HC groups were restricted to action stimuli that we have shown previously to confound imitative- with spatial-compatibility effects (Shaw et al., 2017). For this reason, we question the degree to which responses to these stimuli reflect true mimicry. Since behavior was comparable across clinical and HC groups in response to action stimuli for which this confound was removed, we suggest that MPD and AD patients were expressing difficulties in interference resolution (a cognitive control process through which relevant information is selected and irrelevant information suppressed) rather than imitative control *per se*. To our knowledge, the only study that has investigated imitative tendencies in PD also employed spatially confounded stimuli (Hauschild et al., 2018). As such, the elevated compatibility effect shown by patients with Borderline PD in this former study might also index dysfunctional cognitive control.

Both clinical groups achieved poorer accuracy in visual perspective taking relative to HCs. We are unaware of any previous investigations into this low-level socio-cognitive ability in either PD or AD, and so this finding has two important implications. Firstly, the capacity of this simple experimental task to differentiate between the MPD, AD, and non-clinical groups presents an important novel contribution to the literature on social cognition in PD. Poorer perspective-taking performance may provide an important index of dysfunction to a fundamental cognitive domain in this psychiatric population; patients with Avoidant PD demonstrate difficulties in suppressing their own perspective in order to adopt that of another (“decentration”; Dimaggio et al., 2009; Moroni et al., 2016), and those with Borderline PD express problems in distinguishing between internal psychological content and objective reality (“differentiation”; Semerari et al., 2014, 2015). In this light, perspective taking in PD might reveal dissociable patterns of impairment to self-other distinction (SOD); when faced with conflicting self- and other-representations, patients with MPD appear to default to an egocentric self-bias while the hyper-mentalizing observed in Borderline PD suggests the reverse altercentric bias (Semerari et al., 2014). Such alterations to SOD may be another manifestation of dysfunctional cognitive control; namely, an inability to switch flexibly between cognitive self- and other-representations.

Second, similarly poorer perspective-taking ability in the AD group might indicate the role of altered emotionality in this socio-cognitive impairment in MPD. This finding converges with previous research into disorders characterized by heightened negative emotionality; anxious individuals tend to rely on their own (egocentric) perspective when they attempt to infer what others can see and know (Todd et al., 2015). This may be related to the altered attributional styles expressed by AD patients, who demonstrate negative and depressive biases when interpreting ambiguous events (see Plana et al., 2014). In a similar vein, non-clinical dysphoric individuals exhibit difficulties in taking others’ viewpoints relative to participants without elevated depressive symptomatology (Nilsen and Duong, 2013). Further, major depression has been associated with a negative interpretative bias when processing facial expressions (heightened sensitivity toward sad relative to happy facial expressions; Weightman et al., 2014 [but see Ferguson and Cane, 2017]). Perhaps, then, anxiety and depressive symptoms included in the clinical characterization of MPD might exert an adverse influence on SOD. Further research is needed to elucidate the potential contribution of these comorbidities in dysfunction to cognitive control in PD. Future studies should consider employing additional (independent) measures of anxiety and depression, which might provide insights into their potentially moderating influence.

We observed comparable expressions of affective empathy among all clinical and non-clinical groups in both the self-report and performance-based measures. Although this may be surprising at first glance, inconsistencies in the existing literature suggest that any impairments to empathic expression are subtle, complex, and highly variable; while some studies report disturbances to affective but preserved cognitive empathy (Ritter et al., 2011), others have found the opposite pattern (Cain et al., 2015) or highlight the remarkable heterogeneity present even within discrete diagnostic groups (Baskin-Sommers et al., 2014). Both clinical groups reported significantly higher personal distress when empathizing, however, indicating disruption to the process through which we attribute the source of one’s own emotional state to the target of our empathic expression. In light of the aforementioned results, we suggest that such misattributions stem from dysfunctional SOD arising from long-term elevations of negative emotionality.

This leads us to our observation that both clinical groups reported poorer self-regulation of their emotions – the MPD group in highly demanding situations, and AD participants following failure. When faced with challenging events, these individuals tend to display stronger state orientation – a regulatory mode characterized by indecision and hesitation that prevents change to mental and behavioral states (Kuhl, 2000). Dispositions toward state orientation in these clinical populations is unsurprising given the difficulties they exhibit in down-regulating negative and up-regulating positive emotions; poor regulatory control over one’s emotional reactions has long been recognized as a reliable predictor of many psychiatric (affective) illnesses, and emotional inflexibility predicts pathology following stressful events (Coifman and Summers, 2019). Moreover, the results of the present study align closely with those of our previous investigation with an independent large-scale sample, which revealed an inflexible personality profile characterized by a strong tendency toward avoidant, borderline and depressive personality styles, and state-oriented regulation (Shaw et al., 2018). This propensity to remain in a state-oriented regulatory mode has been associated with poor self-access and discrimination between one’s own and others’ thoughts, wishes, and expectations, especially under highly demanding and stressful conditions (Koole et al., 2006). Our own and others’ research support these assumptions: state- relative to action-oriented individuals reported weaker motivation and poorer ability to adopt the perspective of others, together with higher personal distress when empathizing (Shaw et al., 2018); and patients with Avoidant PD show disturbances in the identification of their own inner states (“monitoring”; Semerari et al., 2014; Moroni et al., 2016). In this light, it would be very informative to determine how such ability to recognize and label one’s emotions, contributes to the socio-cognitive profile revealed here. Unfortunately, our test battery did not include any measure focusing on self-awareness, and so this question remains open for the time being.

In summary, this study has revealed that patients with MPD exhibit a profile of disturbances to emotion recognition, interference resolution and perspective taking, together with heightened distress when empathizing with others and a disposition toward state-oriented emotional regulation. We have interpreted this profile as an indication of compromised cognitive control, resulting in poor performance on measures of social cognition that require the monitoring, updating and shifting among cognitive representations, and inhibition of inappropriate responses. Indeed, there is growing awareness of the role played by these executive functions in social cognition (Binney and Ramsey, 2019; Darda et al., 2019). Such dysfunction in cognitive flexibility would also contribute to the maladaptive disposition toward state-oriented emotional regulation exhibited by the MPD group. This combination of cognitive inflexibility and a stronger tendency toward state orientation could lead to both the attributional biases and inefficient self-other distinction demonstrated by these patients, which together could underlie the dysfunctional interaction styles characteristic of PD, especially in negatively valenced and challenging situations.

It is important to acknowledge several potentials limitations with this study that should be addressed in future research. First, the modest size of our patient groups will have suppressed the statistic power of this experiment. Moreover, since the population of patients with MPD is characterized by considerable heterogeneity, it is unclear whether our results generalize to other samples. Consequently, although our findings of socio-cognitive disturbances in MPD converge with research into other PD samples, our interpretations should be treated with caution until these preliminary results are replicated in much larger samples. Second, given the length of our test battery for social cognition, we chose not to extend it further with additional measures of general executive abilities. Evidence suggests that socio-cognitive capacities are related to more general executive functions, however (Qureshi and Monk, 2018), and that PD is associated with executive dysfunction (Coolidge et al., 2004). Future research is needed to investigate how executive *dys*function might be associated with, or even drive the impairments to social cognition that we have observed in the present study. Finally, existing evidence indicates the importance of symptom severity in understanding socio-cognitive disturbances in this patient population (Semerari et al., 2014, 2015; Moroni et al., 2016), but global psychopathology was not measured in the present study protocol. It is unknown, therefore, whether any of the disturbances to social cognition we have revealed are associated with indices of symptom severity, such as global severity index. Future research should build on our findings to address this.

Notwithstanding these limitations, this study provides a strong basis for subsequent research into social cognition in a patient population that is largely overlooked yet prevalent in clinical practice. By investigating multiple components of social cognition rather than single socio-cognitive ability, and using performance-based measures of both elementary and higher-level socio-cognitive abilities, future research that employs similar test battery might identify the social cognitive deficits that drive the atypical interpersonal behavior of MPD. Performance-based measures are likely to capture different aspects of socio-cognitive abilities to which self-report instruments are insensitive (i.e., their rapid moment-by-moment execution during real-world reciprocal social interactions). Our study also demonstrates that the assessment of social cognition does not have to be time consuming – similar test batteries could be administered to screen for socio-cognitive abilities in clinical practice to complement information collected via questionnaires on metacognition.

## Data Availability Statement

The datasets generated for this study are available from the corresponding author KC upon reasonable request.

## Ethics Statement

The studies involving human participants were reviewed and approved by The Ethics Board of the Institute of Psychology, Academy of Sciences in the Czech Republic. The participants provided their written informed consent to participate in this study.

## Author Contributions

KC and DS: experimental design, behavioral data analyses, and manuscript preparation. ZP: clinical evaluation, data collection, and manuscript preparation. MB: experimental design and manuscript preparation.

## Conflict of Interest

The authors declare that the research was conducted in the absence of any commercial or financial relationships that could be construed as a potential conflict of interest.

## Footnotes

1 http://www.vislab.ucl.ac.uk/cogent.php
